# The Dilemma of Use of Anticoagulation in Patients With Heart Failure With Reduced Ejection Fraction and Sinus Rhythm: A Case Report and Literature Review

**DOI:** 10.7759/cureus.35211

**Published:** 2023-02-20

**Authors:** Muhammad Haseeb ul Rasool, Dhandevi Persand, Sanna Salam

**Affiliations:** 1 Medicine, Icahn School of Medicine at Mount Sinai, Queens Hospital Center, New York City, USA; 2 Internal Medicine, Icahn School of Medicine at Mount Sinai, Queens Hospital Center, New York City, USA

**Keywords:** warfarin, left ventricle (lv) thrombus, systemic anticoagulation, sinus rhythm, heart failure with reduced ejection fraction

## Abstract

Heart failure results in significant morbidity and mortality. Heart failure with reduced ejection fraction (HfrEF) in the absence of atrial fibrillation has been increasingly considered an independent risk factor for ischemic stroke, partly because of the development of left ventricular thrombus and subsequent cardioembolic stroke and partly because of hemodynamic impairment. Here, we present a case of a 60-year-old male with heart failure with reduced ejection fraction, who presented with cardioembolic ischemic stroke. In the investigation to localize the source, he was found to have slow intra-ventricular blood flow, which over shorter periods of follow up lead to the development of left ventricle intra-mural thrombi. Meanwhile, the patient also developed hemorrhagic conversion in the ischemic stroke, which further complicated the choice of anticoagulation. To date, no consensus has been developed on the choice of anticoagulation and clinical criteria for the use of anticoagulation in patients having HfrEF and sinus rhythm. This case brings forth a need for further research on whether anticoagulation would be beneficial in patients with HfrEF and sinus rhythm.

## Introduction

Heart failure is a complex clinical syndrome with rising prevalence worldwide, described by either systolic or diastolic dysfunction. Heart failure is the cause of a significant risk of mortality and morbidity. A number of comorbidities including diabetes, hypertension, hyperlipidemia, coronary artery disease, and peripheral artery disease co-exist with heart failure and complicate the disease’s natural disease pathway. Among the various methods to classify heart failure, the most common method of classification is based on the left ventricular ejection fraction (LVEF), constituting three major categories: heart failure with preserved ejection fraction with LVEF >50%, heart failure with reduced ejection fraction (HFrEF) with LVEF <40%, and heart failure with mid-range ejection fraction (HFmrEF) with LVEF 40-49.9% [1].

HFrEF is being increasingly associated with a higher risk of ischemic stroke, even in the absence of atrial fibrillation. This is attributed to endothelial dysfunction and intra-ventricular stasis leading to the formation of microthrombi. In a population-based cohort study spanning over a duration of 30 years, it was concluded that patients who have heart failure have an increased one and five-year risk of developing ischemic stroke (1.4% and 3.9%), intracranial hemorrhage (0.2% and 0.5%), and subarachnoid hemorrhage (0.03% and 0.07%) [2]. A similar study performed by Berger et al. in 2019 showed that patients with HFrEF without atrial fibrillation have an incidence rate of 1.91 as compared to patients without heart failure and atrial fibrillation [3]. Kondo et al. conducted a pooled patient-level cohort of PARADIGM-HF, ATMOSPHERE, and DAPA-HF trials to devise a formula based on plasma-B-Type natriuretic peptide level, insulin-dependent diabetes, and prior history of stroke, which was validated to calculate the risk of stroke in patients with heart failure and no atrial fibrillation [4]. It was demonstrated that based on the risk factors, for patients in the higher quintile, the risk of developing stroke was 21.2 per 1000 patients-years, which is similar to the risk of stroke for patients with atrial fibrillation not receiving anticoagulation. A reciprocal relationship between ischemic stroke and heart failure was found by Ravandi et al [5]. It was found that among patients with ischemic stroke, 30.0% had either HFrEF or HFmrEF. The incidence of heart failure was significantly higher in patients with a history of hypertension or myocardial infarction [5]. Therefore, optimization of heart failure therapy and compliance is necessary to reduce the risk of the development of ventricular thrombus and/or microemboli that can lead to stroke.

To date, only a few studies have been done to provide guidelines on the use of anticoagulation for patients with HFrEF with sinus rhythm for primary and secondary prevention of ischemic or embolic stroke. Most of these studies have been equivocal on the evidence to support the use of anticoagulation. Therefore, no consensus exists on if whether these patients should be on prophylactic anticoagulation, or when the anticoagulation should be initiated. We present an interesting case of a male who developed an ischemic stroke while having HFrEF and sinus rhythm.

## Case presentation

A 60-year-old male with past medical history of hypertension, hyperlipidemia, type 2 diabetes, non-ischemic cardiomyopathy due to hypertension, and heart failure with reduced ejection fraction (EF: 30%) presented with headache, blurry vision, nausea, and non-bilious emesis that started 18 hours before the time of presentation to the emergency department. On the physical exam, the patient was found to be alert and oriented, with bilaterally symmetrical pupils, minimal reaction to light, and a right temporal homonymous hemianopia. The patient had bilaterally equal and comparable muscle power, intact facial symmetry, and no tongue deviation. There was no pronator drift, reflexes were bilaterally symmetrical, and toes were bilateral down-going.

**Figure 1:**
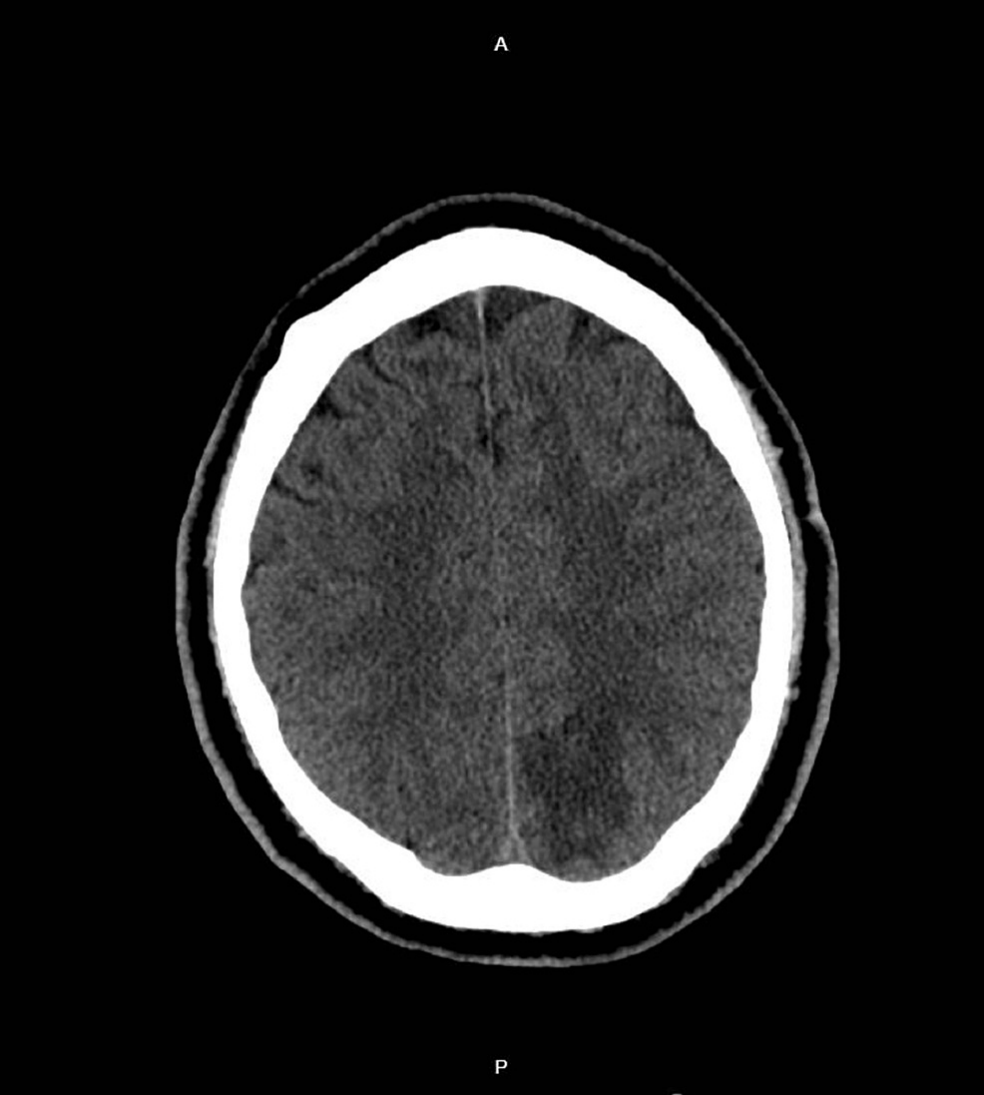
CT brain showing left occipito-parietal cortex hypodense area with loss of gray-white matter differentiation, suggestive of evolving stroke CT: Computerized tomography

**Figure 2:**
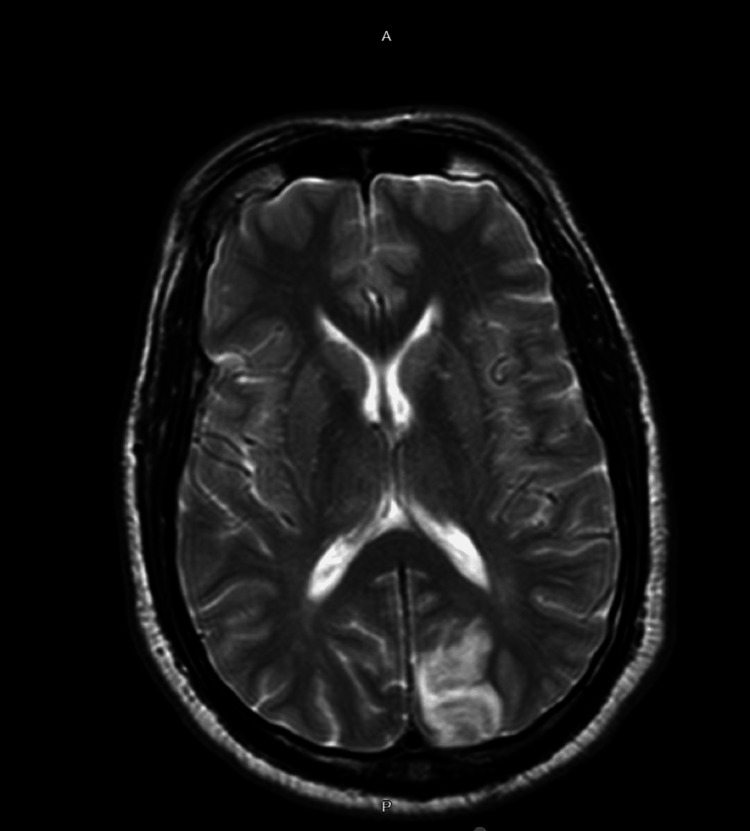
MRI brain T2W images showing left occipital lobe ischemia with a hemorrhagic component MRI: Magnetic resonance imaging; T2W: T2 weighted

Computerized tomography (CT) head showed an area of hypodensity in the left parieto-occipital region with loss of gray-white matter differentiation suggestive of evolving stroke within posterior cerebral artery territory (Figure 1). Computerized tomography angiogram head and neck showed atretic, but patent left vertebral artery or right vertebral artery dominance and did not show any significant large vessel stenosis or occlusion. The patient was given aspirin 325 mg and Plavix 300 mg and was started on a high-dose statin. The patient was deemed not a suitable candidate for thrombolysis because he was out of the therapeutic window for intervention. Magnetic resonance imaging (MRI) brain showed a new wedge-shaped 4 cm region of serpiginous diffusion restriction within the left occipital lobe favoring left occipital lobe ischemia with hemorrhagic component (Figure 2). CT head without contrast was performed to confirm the hemorrhagic component, which was consistent with the MRI findings. As a part of a stroke work assessment, the lower extremity venous duplex was performed which showed no evidence of deep venous thrombosis.

An interval CT head performed 48 hours after the onset of symptoms showed an area of abnormally low attenuation consistent with encephalomalacia in the left posterior parietal and superomedial occipital lobe. There was no shift of midline structures or mass effect. No acute hemorrhage, hydrocephalus, or any additional interval changes of significant pathology were seen. An electrocardiogram showed sinus rhythm with 85 beats per minute, left atrial enlargement, and left axis deviation with left ventricular hypertrophy. Blood workup performed for the hyper-coagulable state was negative. 

An echocardiogram was performed which showed mild left ventricular hypertrophy, LV ejection fraction of 30%, grade I diastolic dysfunction, moderately dilated right atrium, right ventricle, left atrium, and aortic root. The right ventricle was moderately hypokinetic. There was pan-valvular mild regurgitation. The inferior vena cava was normal in size with more than 50% respiratory excursion. The left ventricle was moderately dilated with left ventricle hypertrophy and diffuse hypokinesis with an ejection fraction of 30%. The echocardiogram with contrast was performed to rule out intra-ventricular thrombus, which showed severely diffuse left ventricular hypokinesis. Though there was no definite intraventricular thrombus, the flow at the apex was found to be extremely sluggish, posing a high risk to develop microthrombi. 

**Figure 3:**
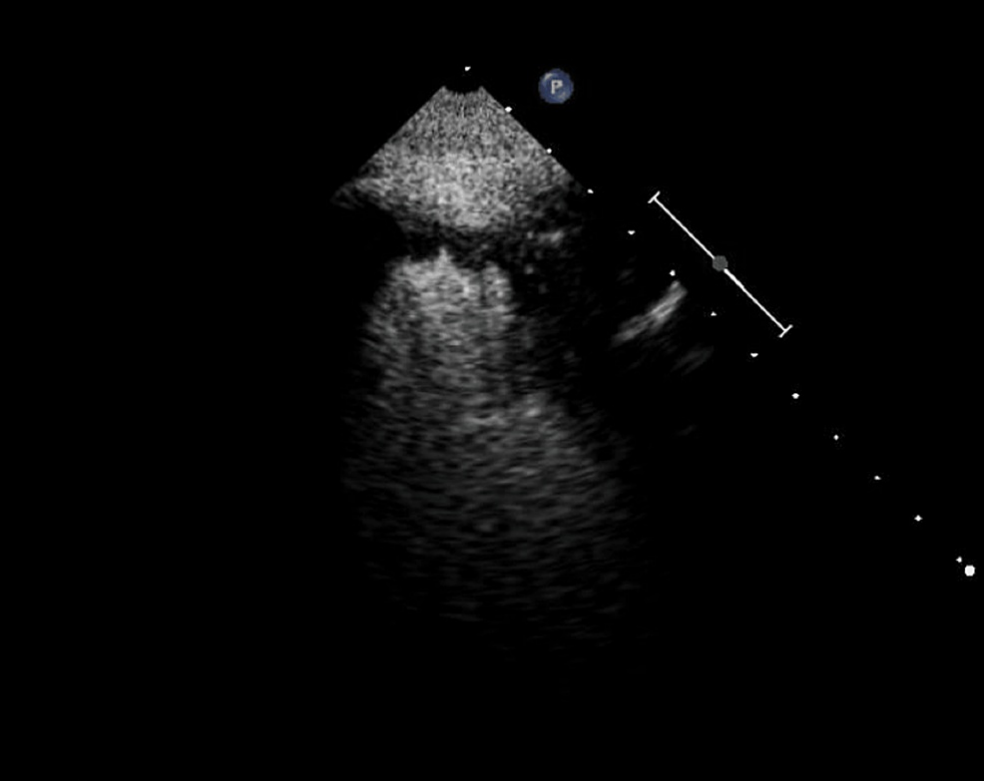
Echocardiogram with contrast showing 6x6mm mobile ventricular thrombus

The patient was continued on aspirin only, while clopidogrel was held according to the recommendation of neurology. After completion of the first 48 hours of permissive hypertension, blood pressure was normalized. The interval physical exam after 72 hours of the presentation showed bilaterally symmetrical pupils and a restricted right visual field; however, the patient gained 10-25 degrees of vision from the midline. The patient had a risk of paradoxical emboli score of +5, which translates to a 34% chance of paradoxical cardio-embolism. Considering the risk, a repeat echocardiogram with contrast was performed which showed severe diffuse left ventricular hypokinesis and a small mobile thrombus in the left ventricular apex that was 6x6mm (Figure 3). Based on the findings of ventricular thrombus in the setting of a recent hemorrhagic stroke, neurology and cardiology teams jointly recommended that the patient be started on warfarin for secondary prevention of stroke in view of the history of heart failure with reduced ejection fraction with a target goal INR of 2.0-3.0. The patient regained the vision in the lost right hemifield 72 hours post-stroke. The patient is being closely followed in the outpatient neurology and cardiology clinic.

## Discussion

Patients with heart failures are divided into three categories based on the LVEF. Those with documented LVEF <40% are categorized as HfrEF. It is estimated that in 2015, over 5.7 million Americans had heart failure, out of which 2.6 million had reduced ejection fraction. Approximately 1.9 million of these people have sinus rhythm [6]. A retrospective study at Brigham and Women’s hospital examining precipitating factors of LV thrombus on echocardiography found that heart failure was present in 68.5% of cases and was the most common precipitating factor [7]. The enhanced risk of cerebrovascular accidents with heart failure was first coined by the Framingham Study in 1983 and is well appreciated in medical literature but not well managed [8].

 It is postulated that heart failure negatively affects all three components of Virchow’s Triad including impaired blood flow, hypercoagulability, and endothelial dysfunction. The increased risk of stroke in heart failure is attributed to both embolic and hemodynamic phenomena. Regarding the embolic phenomenon, it has been proposed that severely impaired LV contractility can form intra-mural thrombi, endothelial dysfunction can lead to the development of atherosclerotic plaques in supra-aortic vessels, and clinically unapparent atrial fibrillation can increase the risk of formation of thrombi in left atrial appendage. While these proposed avenues of thrombogenesis can be targeted with vitamin K antagonists and non-vitamin K oral anticoagulants (NOACs), the impaired hemodynamics due to heart failure can also play a substantial role in cerebral hypoperfusion, which cannot be addressed by the use of anticoagulation [9]. 

 Multiple randomized trials have demonstrated that reduced LV ejection fraction is inversely related to increased mortality and cardiovascular events. Candesartan in Heart Failure Reduction in Mortality (CHARM) trial demonstrated that every 10% reduction in LVEF below 45% results in 39% increased all-cause mortality, whereas a similar relationship is lost after LVEF above 45% [10]. However, the relationship of reduction in LVEF with the incidence of thromboembolic events has been questioned, where equivocal evidence for refuting and suggesting the association has been documented [11]. In the Warfarin versus Aspirin in Reduced Ejection Fraction (WARCEF) trial, the safety of the use of warfarin and aspirin was compared, and data were incremented with a reduction in LVEF. Incidence of heart failure hospitalization and death either from the cerebrovascular event, all-cause mortality, or sudden cardiac mortality was considered as the endpoint. It was calculated that the incidence of the primary endpoint was similar between the aspirin and warfarin groups. However, when the stroke was used as the primary outcome, the risk of stroke was 0.72% per year in the warfarin arm, as compared to 1.36% per year risk in the warfarin arm. Additionally, treatment with warfarin was found to be associated with significantly greater stroke risk over the incremental decline in LVEF (HR: 1.346, 95% CI 1.044, 1.737; p=0.022) than those treated with aspirin (HR: 0.971, 95% CI 0.805, 1.171; p=0.757; p-value for the interaction=0.04); therefore, in patients with declining left ventricular ejection fraction despite being on maximal goal-directed medical therapy, use of warfarin can cause more harm than benefit [12]. 

Contrary to the prior findings, the Warfarin/Aspirin Study in Heart Failure (WASH) found that there was no evidence of aspirin being safe or effective in patients with heart failure. The study also could not demonstrate the benefit of the use of warfarin for patients with HFrEf with sinus rhythm [13]. Heart failure Long-Term antithrombotic study (HELAS) was a randomized, placebo-controlled trial to demonstrate the effect of long-term antithrombotic therapy in patients with chronic heart failure. Patients with ischemic heart disease were randomized to receive aspirin or warfarin. Patients with dilated cardiomyopathy were randomized to receive either warfarin or a placebo. Endpoints used in the study were myocardial infarction, hospitalization, exacerbation of heart failure, death, and hemorrhage of clinical significance. It was concluded that there was no significant difference in the different groups with regard to the primary endpoint and the treatment had no effect on the outcome. There were no reported incidences of peripheral or pulmonary emboli [14]. 

The Warfarin and antiplatelet therapy in chronic heart failure (WATCH) trial was performed with the intent to determine the optimal antithrombotic agent for HFrEF and sinus rhythm. Patients were randomly allocated to aspirin 162 mg daily, clopidogrel 75 mg once daily, and warfarin with a target INR of 2.5-3.0. Occurrences of death, non-fatal stroke, and non-fatal myocardial infarction were the endpoints of the study. For the primary composite endpoint, the hazard ratio for warfarin versus aspirin was 0.98 (95% CI: 0.86 - 1.12; P=0.77); for clopidogrel versus aspirin was 1.08 (95% CI: 0.83-1.40; P=0.57); and for warfarin versus clopidogrel was 0.89 (95% CI: 0.68 - 1.16; P=0.39). Patients treated with warfarin were found to have fewer non-fatal strokes than aspirin or clopidogrel. Heart failure-related hospitalization occurred in 116 (22.2%), 97 (18.5%), and 89 (16.5%) patients treated with aspirin, clopidogrel, and warfarin, respectively (P=0.02 for warfarin versus aspirin). It was concluded that neither warfarin is superior to aspirin, nor clopidogrel is superior to aspirin with regard to mortality data [15]. 

 The risk of stroke in a high-risk cohort of patients has been calculated to be 1.9% annually, which is similar to the risk for patients with atrial fibrillation and CHA2DS2-VASc score of 2, for which anticoagulation with warfarin has been found to be clinically effective and recommended. However, a major limitation of the evidence of the use of anticoagulation is the lack of including the bleeding risk as part of the analysis making the risk-benefit assessment difficult [16]. In another analysis performed by Eckman and the team, it was found that for the non-warfarin oral anticoagulants, the stroke risk threshold can be as low as 0.9% per year for clinical benefit. [17]. A sub-study of WARCEF trial data demonstrated that the use of higher-quality anticoagulation, reflected by the time-in-therapeutic range is associated with better clinical outcomes [18]. NOACs have demonstrated more consistent anticoagulant effects with lesser monitoring; however, no randomized clinical trial has been done to date to demonstrate their effectiveness as compared to aspirin and warfarin. Considering the fact that major strokes have been graded as worse than death on the death on quality-of-life index scales, there is a need to develop randomized clinical trials to evaluate the effects of NOACs as compared to warfarin, in patients with HFrEF, while including the risk of bleeding to provide clinical evidence for risk-benefit discussion. 

## Conclusions

HFrEF with sinus rhythm is a well-appreciated risk factor for ischemic cerebrovascular disease; however, literature has equivocal evidence regarding the use of anticoagulation and which agent is superior to the rest. A significant number of patients presenting with stroke are found to have heart failure, reflecting a considerable cohort of the population for which no clinical guidelines are available. This case report highlights the importance of conducting prospective placebo-controlled clinical trials in such cases to help identify the correct treatment modality and duration.
